# Breast cancer intra-tumor heterogeneity

**DOI:** 10.1186/bcr3658

**Published:** 2014-05-20

**Authors:** Luciano G Martelotto, Charlotte KY Ng, Salvatore Piscuoglio, Britta Weigelt, Jorge S Reis-Filho

**Affiliations:** 1 Department of Pathology, Memorial Sloan Kettering Cancer Center, New York, NY, 10065, USA

## Abstract

In recent years it has become clear that cancer cells within a single tumor can display striking morphological, genetic and behavioral variability. Burgeoning genetic, epigenetic and phenomenological data support the existence of intra-tumor genetic heterogeneity in breast cancers; however, its basis is yet to be fully defined. Two of the most widely evoked concepts to explain the origin of heterogeneity within tumors are the cancer stem cell hypothesis and the clonal evolution model. Although the cancer stem cell model appeared to provide an explanation for the variability among the neoplastic cells within a given cancer, advances in massively parallel sequencing have provided several lines of evidence to suggest that intra-tumor genetic heterogeneity likely plays a fundamental role in the phenotypic heterogeneity observed in cancers. Many challenges remain, however, in the interpretation of the next generation sequencing results obtained so far. Here we review the models that explain tumor heterogeneity, the causes of intra-tumor genetic diversity and their impact on our understanding and management of breast cancer, methods to study intra-tumor heterogeneity and the assessment of intra-tumor genetic heterogeneity in the clinic.

## Introduction

Intra-tumor heterogeneity denotes the coexistence of subpopulations of cancer cells that differ in their genetic, phenotypic or behavioral characteristics within a given primary tumor, and between a given primary tumor and its metastasis. This diversity can be attributed to genetic and epigenetic factors, and to non-hereditary mechanisms such as adaptive responses or fluctuation in signaling pathways [1,2]. Cancer cells within a given tumor may differ not only phenotypically or in relation to genetic aberrations that do not confer an overt phenotype, but also in terms of their driver genetic aberrations. For instance, HER2 status may vary between primary tumors and their respective metastasis or circulating tumor cells (CTCs) [3,4], and examples of primary invasive breast cancers containing neoplastic cells with and without *HER2* amplification are on record [5]. Thus, intra-tumor heterogeneity poses a tremendous challenge for the characterization of biomarkers and treatment selection.

Massively parallel sequencing (MPS) studies of breast cancers and other tumor types have shown that spatial and temporal heterogeneity are common phenomena [6-11]. Thus, biopsies of a small tumor region may not provide a representative characterization of the genetic, epigenetic and/or phenotypic alterations found in the tumor as a whole [6,12]. It is now apparent that further complexity is added to the challenges posed by intra-tumor genetic heterogeneity when dimensions of space and time are incorporated to reflect the dynamic nature of tumors [13]. New insights into the underlying features, mechanisms and consequences of tumor heterogeneity and new approaches to characterizing intra-tumor genetic heterogeneity are crucial for the improvement of existing therapies and for the realization of the potential of precision medicine.

## Tumor heterogeneity

The theories describing the establishment and maintenance of tumor heterogeneity are the cancer stem cell (CSC) hypothesis [14] and the clonal evolution/selection model [15], two concepts that were initially thought to be mutually exclusive, but are now perceived as potentially complementary [2,16]. Both concepts consider that tumors originate from single cells that have acquired multiple molecular alterations, have developed indefinite proliferative potential, and assume that (micro)environmental cues have an impact on the composition of a cancer. These theories, however, have fundamental differences (Table 1); while the CSC hypothesis attributes heterogeneity to aberrant differentiation programs and presupposes the existence of a hierarchical organization of cancer cells, the clonal evolution model explains intra-tumor diversity as speciation by natural selection and does not rely on a hierarchical model. Importantly, the CSC hypothesis states that only a small fraction of cells can drive tumor progression and are inherently therapy-resistant. For the clonal evolution model, progression and resistance to therapy would follow Darwinian evolutionary rules, where the emergence of clones that would be able to progress or be resistant to a given therapy would depend on the cell population size, mutation rate (that is, genetic instability), proliferation rate and selective pressures imposed by the (micro)environment and/or external selective pressures (for example, therapies) [17].

**Table 1:** Tenets of the cancer stem cell and clonal evolution models

	**Cancer stem cell model**	**Clonal evolution model**
Tumorigenic cells	CSCs	Any cell
Tumor cell organization	Hierarchical	Stochastic
Capacity of self-renewal with asymmetric divisions	CSCs can self-renew indefinitely whereas terminally differentiated cells have limited proliferative potential	Not applicable
Progression	Driven by CSCs, which account for a small subpopulation of the tumor bulk	Driven by the fittest clone under a constellation of selective pressures
Source of heterogeneity	Aberrant differentiation program and mutations	Epigenetic and genetic aberrations followed by selection
Type of heterogeneity	Initially perceived as largely phenotypic; however, more recent studies suggested that CSCs may be genetically heterogeneous within a tumor	Genetic and phenotypic heterogeneity
Source of resistance to therapy	CSCs	Selection of resistant subclones harboring specific genetic or epigenetic aberrations

CSC, cancer stem cell.

## The cancer stem cell hypothesis

The CSC concept proposes that, within a given tumor, a phenotypic hierarchy exists, with a minor subset of the so-called CSCs at the apex and highly proliferating, lineage-committed progenitors and terminally differentiated cells at the base [18]. One of the tenets of this model is that tumor growth, disease progression and the generation of heterogeneity in cancers are driven by a small population of tumorigenic cells within a tumor, whilst the vast majority of cancer cells do not contribute to tumor growth and would be unable to repopulate a tumor after a given tumor cell ablative therapeutic intervention [14]. Hypothetically, CSCs can self-renew indefinitely, drive tumor growth and differentiate into virtually all cell types found in a tumor [14], thereby spawning heterogeneity. On the other hand, progenitors and terminally differentiated cells are highly proliferative, display lineage commitment, and have limited proliferative potential (that is, finite number of cell divisions) and little or no capacity to contribute to disease progression [14]. While the CSC model does not tackle the question regarding the cell of origin, in particular whether cancers arise from normal stem cells, it proposes that many cancers may be hierarchically organized [19].

CSCs are described operationally, by challenging cancer cells for their ability to form tumors in a relatively permissive environment (that is, immunosuppressed mice) [14]. The identification of the population able to form tumors under these settings is often defined on the basis of cell-surface markers, sometimes derived from the analysis of normal stem cells in the tissue of origin. In the case of breast cancer, the existence of stem-like cells was inferred in a study demonstrating that as few as 100 CD44^+^CD24^-/low^ breast cancer cells could efficiently form tumors when injected into mice [20], whereas the efficiency of cells of other phenotypes was non-existent or significantly lower. It was clear, however, that the CD44^+^CD24^-/low^ surface markers enrich for tumorigenic cells in some, but not all, breast cancers [20]. The validity of the combination of these markers as a definition of breast CSCs has been called into question [21], and additional markers have been reported (for example, ALDH1 [22]).

The tenets of the CSC hypothesis are being disputed, given the evidence demonstrating the existence of a dynamic equilibrium between differentiated cells and CSCs [23], whereby not only CSCs can differentiate into terminally differentiated cells, but terminally differentiated cells can also de-differentiate into a CSC state, and the overlap between some phenotypic characteristics of CSCs and the phenomenon of epithelial-to-mesenchymal transition. Hence, in some contexts, the CSC phenotype may represent a state that cancer cells within a tumor can acquire rather than a discrete population of cancer cells that constantly display those properties. Additionally, it is clear that the definition of CSCs is assay-dependent and may not accurately reflect the true physiology in humans [24]. Xenotransplantation assays applied to breast cancer may only identify cells capable of engraftment in particularly permissive (micro)environments. In addition, there is now direct evidence to demonstrate that at least in some types of cancer (that is, acute lymphoblastic leukemia) intra-tumor genetic heterogeneity is found not only in terminally differentiated cells, but also in the CSC population as defined by xenograft experiments [25]. For a detailed review on the CSC hypothesis and its limitations, readers are referred to Meacham and Morrison [14].

The CSC hypothesis and the extensive intra-tumor genetic heterogeneity observed in some tumors (see below) are not trivially reconciled. For instance, in tumors with high degree of genetic heterogeneity, phenotypic and/or functional differences between cells may reflect variations at the genetic level rather than a CSC differentiation hierarchy [14]. Therefore, integrating tumorigenic potential and genetic heterogeneity studies is germane for a more representative picture of the extent to which individual tumorigenic cells contribute to genetic diversification.

## The clonal evolution model

The clonal evolution model posits that although most neoplasms arise from a single cell, cancer cells have varying degrees of genetic instability and acquire additional genetic aberrations during tumorigenesis and tumor evolution. This process leads to the development of a cancer cell population that, albeit clonal in origin, is composed of multiple subpopulations that, in addition to the founder genetic events, harbor private genetic aberrations. In this model, the modal clone (that is, the most frequent clone) in the tumor cell population is defined by the tumor cell characteristics and the constellation of selective pressures it is subjected to. A simplistic model of clonal evolution is portrayed as a succession of clonal expansions, where each round is driven by the acquisition of additional random mutations [2,13] that can be deleterious, neutral or confer a biological advantage (that is, proliferation and survival). The latter may result in clonal expansion [15]. It is important to note that not all clonal expansions may be triggered by genetic events; epigenetic mechanisms, such as DNA methylation, histone modification, nucleosome positioning, and microRNA expression affecting the regulation of gene expression or creating permissive characteristics that would result in a substantial increase in fitness of a given clone, may also play a role.

This linear path of clonal expansions is arguably simplistic and only conveys the key mutational events that drive progression, namely 'driver' mutations. The acquisition of such mutations, however, is often accompanied by 'passenger' alterations (hitchhiker mutations equivalent in evolution theory) that seem to confer no selective advantage or have no phenotypic manifestation under a given set of selective pressures [15]. Importantly, a given mutation can change from a passenger to a driver aberration should the selective pressures change. In addition, epistatic interactions may result in phenotypes that differ from those inferred by the sum of the phenotypes caused by each of the mutations [26]. As tumors progress, the mutational rate may vary [27], leading to genetic diversification within the tumor as the clones harboring new mutations that are neutral and some of the less-fit clones may be produced more rapidly than they are eliminated [15,27]. Tumors with higher genetic complexity (that is, with a larger variety of clones harboring private mutations or more private mutations in each clone) have a greater variety of genetic aberrations to be subjected to selective pressures, and, consequently, the probability of the existence of a clone that can be fit under a new set of selective pressures is greater than in tumors with low levels of intra-tumor genetic heterogeneity [2].

## Intra-tumor genetic heterogeneity in breast cancer

Darwinian evolutionary rules appear to govern the somatic changes that constantly occur within a tumor. With the advent of MPS it has become possible to establish the existence of intra-tumor genetic heterogeneity within a primary tumor and between a primary tumor and its metastasis. The seminal study by Gerlinger and colleagues [6] has elegantly demonstrated both phenomena in kidney cancers. In breast cancer, evidence of intra-tumor genetic heterogeneity has been documented by cytogenetic analysis, chromosomal and microarray-based comparative genomic hybridization (CGH) and, more recently, MPS analysis [5,7,9-11,17,28-32]. Importantly, a recent study by Shah and colleagues [11] has demonstrated that the intra-tumor genetic heterogeneity found in breast cancers may affect even known driver genetic aberrations, such as *TP53* and *PIK3CA* somatic mutations. Furthermore, there is also evidence to demonstrate that, in individual breast cancers, subclonal populations of cancer cells may exist across different geographical regions of a tumor (spatial heterogeneity) or evolve over time between the primary tumor and a subsequent local or distant recurrence (temporal heterogeneity) [7,9,12,31].

### Causes of intra-tumor genetic heterogeneity

Evolutionary trajectories of tumors are determined by population size, mutation rate and selective pressures [17]. Elevated mutation rate (that is, genomic instability) is a common feature in cancers [27]. This instability can be caused by inherited or somatic aberrations in genes that maintain genome instability, or can be caused by extrinsic mutagens such as cigarette smoke, ultraviolet light and chemotherapy [15]. The aberration spectra of cancer cells often reflect the signatures of the mechanism of genomic instability [15]. Genome-wide profiling of somatic mutations in breast cancers revealed substantial variations in the total number of mutations and the type of mutations [32,33], suggesting that the mutational processes that generate these genomic landscapes vary. It was hypothesized that some of these mutational patterns may be associated with homologous recombination DNA repair deficiency or nucleotide-excision repair. Moreover, regional hypermutation of C > T at TpCpX trinucleotides, known as kataegis, has been documented in breast cancers [32]. Hence, cancer initiation and progression are dependent on mutations and chromosomal rearrangements that can accumulate either gradually over time (gradualism) or in a single catastrophic event, such as chromothripsis (punctuated equilibrium) [34].

The interaction of genomic instability and selective pressure results in intra-tumor heterogeneity; selective pressures, however, are not constant, and may vary spatially due to differences in the (micro)environment, changes in endocrine stimuli, and exogenous parameters (for example, therapeutic intervention) [1]. Thus, although most cells within a tumor are thought to have a clonal origin, genomic instability and the dynamic nature of selective pressure can lead to multiple routes of genetic diversification within the adaptive landscapes of tissue ecosystems [15], and can ultimately result in a genetically, epigenetically and phenotypically diversified tumor.

### Spatial heterogeneity

**Figure 1:**
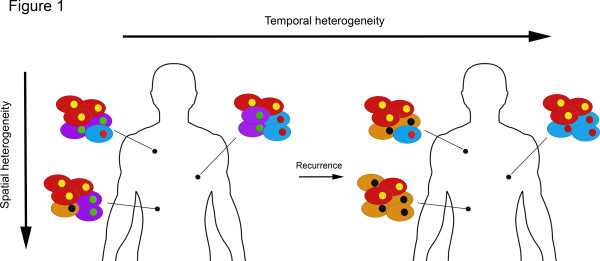
**Spatial and temporal heterogeneity.** Heterogeneity may be present within a given tumor, such that the different regions of the tumor harbor different repertoires of genetic aberrations (spatial heterogeneity), or during the course of disease progression (temporal heterogeneity).

Spatial heterogeneity refers to the genetic variation across different regions within a single tumor. Histological heterogeneity within a tumor is a frequent phenomenon in breast cancers, and one of the defining characteristics of metaplastic breast cancers (Figure 1). In a proof-of-principle study, our group utilized microarray-based CGH, *TP53* sequencing and fluorescence *in situ* hybridization (FISH) to determine the extent of genetic heterogeneity between morphologically distinct areas of individual metaplastic breast cancers [31]. In all but one case, the histologically distinct components of the tumors were shown to be clonal based on the presence of identical *TP53* somatic mutations and similar copy number aberrations. In two cases, however, the morphologically distinct components, albeit clonal, displayed distinct repertoires of gene copy number aberrations, including high-level focal amplifications [31]. These observations are consistent with the notion that morphologically distinct areas of a cancer are at least coincidental with, if not underpinned by, different repertoires of genetic aberrations. Similar observations were made in a case of a triple-negative breast cancer with focal apocrine differentiation [35].

Spatial intra-tumor genetic heterogeneity has also been documented irrespective of differences in the histological features of the areas analyzed. CGH analysis of geographically separate sectors of primary breast cancers, irrespective of the local histological characteristics, has revealed varying levels of genetic heterogeneity within cancers; monogenomic tumors are characterized by the presence of a single major clonal subpopulation with a stable genome, whereas polygenomic cancers harbor multiple genetically distinct subpopulations, which may occupy the same or separate anatomic locations [7]. Similar observations were made by means of single cell sequencing analysis [9]. Interestingly, in two polygenomic breast cancers, subpopulations with increasing genomic complexity followed a linear pattern geographically in the tumor [7] and in one of the two tumors, a subclonal *KRAS* amplification was identified. The tumor cells that harbored the *KRAS* amplification intermixed with those that did not, suggesting that genetically distinct tumor populations do not necessarily segregate spatially [7,9].

### Temporal heterogeneity

There is evidence to demonstrate that tumors evolve over the course of the disease between the primary tumor and local or distant recurrences (Figure 1). Even though genomic analysis of primary breast tumors and their metastases demonstrated their clonal relatedness and revealed similarities in their repertoire of somatic genetic aberrations, differences in their genomes have been systematically documented [28,30,36,37]. While synchronous metastases tend to be largely similar to their corresponding primaries in terms of their repertoire of genetic alterations [30,37], 31% of cases of primary breast cancers and their metachronous metastases revealed significant differences in gene copy number by CGH and FISH [36]. In a more recent study, whole-genome sequencing of the relapse of a lobular breast cancer 9 years after diagnosis revealed that some of the mutations present in 1 to 13% of the cells in the original tumor were enriched in the relapse tumor [10]. In a different report, the primary tumor appeared to have more clonal diversity than the metachronous brain metastasis based on the frequencies of mutations and structural variants, suggesting chemotherapy and/or the microenvironment contributed to the temporal heterogeneity in this case [8]. These observations illustrated that the metastatic lesions share somatic genetic aberrations with their primary tumor, but that their modal populations differ, suggesting that metastases may stem from a single or a limited number of clones from the primary tumor or even from clones present in other metastases rather than from the clones present in the primary tumor [6]. Based on these observations, it is also plausible that the neoplastic populations of primary tumors and their metastases may undergo parallel and independent evolutionary routes.

Another potential temporal evolutionary bottleneck is the progression from *in situ* to invasive disease [17], given that, chronologically, the latter follows the former, in cases where a given *in situ* lesion is the actual precursor of the invasive cancer. Although ductal carcinoma *in situ* (DCIS), a non-obligate precursor of invasive breast cancer, and the infiltrating counterparts appear genetically similar, they occasionally present qualitative differences in phenotypes and genotypes [38,39]. This observation indicates that, despite their clonal relatedness, some invasive tumors may derive from non-modal populations of neoplastic cells within the adjacent DCIS [38]. Moreover, although the modal populations of synchronously diagnosed ipsilateral DCIS and invasive carcinomas are genetically similar, intra-tumor genetic heterogeneity seems to occur early in breast cancer development [38,39], and clonal selection appears to take place during the progression from *in situ* to invasive disease [38,39].

Many therapeutic failures can be attributed to the outgrowth of clones that harbor specific resistance mechanisms and were present before the onset of therapy due to intra-tumor genetic heterogeneity [2,17,26]. For example, gefitinib resistance in patients with *EGFR* mutant non-small cell lung cancers has been shown to be mediated in some cases by the selection of cancer cells harboring the *EGFR*(T790M) gatekeeper mutation and/or *MET* gene amplification [40]. Furthermore, resistance to poly(ADP) ribose polymerase inhibitors and platinum-based chemotherapy in cancer patients harboring a *BRCA1* or *BRCA2* germline mutation is mediated in a proportion of cases by the acquisition of revertant mutations or intra-genic deletions that restore the open reading frame of *BRCA1* or *BRCA2*[41,42]. It is possible, on the basis of the data currently available, that these secondary somatic genetic events affecting *BRCA1* or *BRCA2* may precede the therapeutic intervention [26,42].

## Approaches to characterize intra-tumor genetic heterogeneity

**Figure 2:**
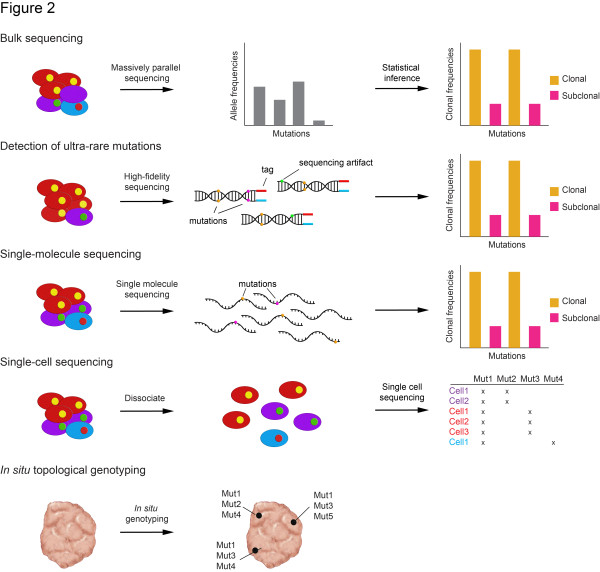
**Approaches to characterize heterogeneity.** Tumor bulk sequencing: massively parallel sequencing of millions of tumor cells can be employed to assess the allele frequencies of mutations. Using statistical methods, the clonal frequencies of these mutations can be inferred. Detection of ultra-rare mutations: mutations that are present in rare populations of cancer cells (that is, comprise <1% of the tumor cell population) can be identified using high-fidelity sequencing, such as allele-specific tagging of DNA molecules, such that only alterations found on both strands are defined as mutations. Single-molecule sequencing: DNA is extracted from tumor cells and sequenced on a single-molecule sequencing platform (for instance, the Pacific Bioscience RS system). Single-cell sequencing: tumors are dissociated into single cells. DNA from single cells is amplified and sequenced using massively parallel sequencing to genotype individual cells. *In situ* topological genotyping: DNA or mRNA is amplified *in situ* on histological sections of the tumor, allowing for the genotyping of the cancer cells within the tumor without losing anatomical and histological information.

Given the clinical implications of intra-tumor heterogeneity, a remaining question is to identify the optimal means to assess this phenomenon, and to monitor spatial and temporal heterogeneity (Figure 2).

### Tumor bulk sequencing

Most cancer genomics studies currently involve the sequencing of DNA extracted from millions of cells of the tumor bulk [6,11,33], which gives the average mutant allele fraction and average allelic copy number. This approach, however, only provides a compound measure of the underlying clonal complexity and the clonal frequencies of mutations and copy number alterations have to be inferred statistically using algorithms such as ABSOLUTE [43] or PyClone [11]. ABSOLUTE jointly estimates tumor purity and ploidy from the observed copy number profiles (and mutational repertoire, if available) accounting for subclonal aberrations [43]. The clonal frequency of a given aberration is then inferred from the estimated tumor purity, ploidy, and local copy number alterations and is classed as clonal or subclonal. PyClone uses a hierarchical Bayesian model to estimate the clonal frequencies of mutations, also accounting for sequencing errors [11]. A Dirichlet process is used to estimate the clonal frequencies, and mutations with similar clonal frequencies are clustered. Although not a definite proof, clustering provides clues to which genetic aberrations may occur together in the same cells [11,44]. These methods are highly sensitive to the accurate estimation of local copy number variation, which is not trivially obtained, as cells within a given tumor do not always have the same copy number alterations, as illustrated by single cell sequencing studies [9]. For both ABSOLUTE and PyClone, however, prior knowledge of the tumors is required to obtain optimal results. For instance, even though ABSOLUTE integrates recurrent cancer karyotype models to identify the most common karyotype that would explain the data, more appropriate results may be obtained by overriding default estimates with prior knowledge on either tumor purity or ploidy. Furthermore, detection of subclonal populations is limited by the error rate of the sequencing platform and aberrations in rare cells may escape detection using standard sequencing depths (100x to 250x coverage). Finally, to infer the clonal composition and architecture of cancers based on bulk sequencing, comprehensive sequencing of the tumor genome is required (that is, currently, at least whole exome sequencing). Despite all these challenges, bulk sequencing remains a popular choice given its cost-effectiveness.

### Detection of ultra-rare mutations

Although standard next-generation sequencing technologies have the capacity to generate hundreds of billions of nucleotides of DNA sequence in a single experiment, the practical limit of detection is imposed by errors introduced during sample preparation and sequencing. For the mainstream MPS platforms such as Ion Torrent and Illumina platforms, the sequencing error rate is reported to range from 0.26% to 1.71% [45]. These scattered sequencing mistakes become very problematic when studying mutations present in <1% of cells [46]. A variety of improvements in biochemistry, sample preparation and data processing have been developed to enhance sequencing accuracy. For example, a tagging method (SafeSeqS) based on labeling single-stranded DNA fragments has been developed and allows for an observed mutation frequency of <0.001% mutations/base pair [47]. More recently, Schmitt and colleagues [46] developed an approach for tag-based error correction called Duplex Sequencing that greatly reduces errors by independently tagging and sequencing the two strands of a DNA duplex. This method has a theoretical background error rate of less than one error per 10^9^ nucleotides sequenced, allowing the detection of ultra-rare variants in heterogeneous populations. However, nucleotides artifactually introduced during the initial round of PCR amplification cannot be accurately detected as errors, even with a tagging technique, if the artifactual mutation is propagated to all subsequent PCR duplicates [46]. Further developments for the *de novo* identification of mutations present in <1% of cancer cells are eagerly awaited.

### Single-molecule sequencing

Bulk tumor sequencing may also be performed using single-molecule sequencing technologies. Compared to MPS, single-molecule sequencing eliminates the need for PCR amplification, and thus the biases introduced by PCR amplification. This approach requires less starting material, has faster turnaround time, and produces longer reads (3 kb) [48] that are advantageous for identification of genomic rearrangements. Profiling intra-tumor heterogeneity by single-molecule sequencing, however, shares the same challenges as bulk sequencing by conventional MPS, in that it is not a direct measure of heterogeneity and the subclonal architecture can only be inferred. Furthermore, increased read length does not benefit calling subclonal point mutations and is likely only useful in samples with optimal DNA quality. The high sequencing error rate at 13%, compared to 0.26% to 1.71% for the most frequently employed MPS platforms [45,48], is particularly detrimental in the context of heterogeneity, as sequencing error may be mistaken for genuine subclonal mutations. Finally, the throughput of single-molecule sequencing is too low for heterogeneity studies. Further technological developments are required for the successful use of single-molecule sequencing in the characterization of intra-tumor genetic heterogeneity in cancers.

### Single-cell sequencing

Currently, the most objective way to assess heterogeneity is single-cell sequencing. In contrast to bulk sequencing either by MPS or single-molecule sequencing, single-cell sequencing allows for the direct inference of clonal genotypes (that is, identification of the repertoire of genetic alterations in each tumor cell that composes a tumor [44]). In this effort, Navin and colleagues [9] have successfully developed and applied MPS to single cells (that is, single nucleus sequencing), and conclusively demonstrated that many breast cancers are composed of multiple genetically distinct subclones. Currently, the costs and time required for whole exome or whole genome single cell sequencing of tumors are prohibitive for clinical use. Furthermore, the relevance of single cell sequencing methods in the diagnostic arena is still unclear since sequencing data derived from single cells do not provide any direct information on the remaining tumor cell population. In addition, whilst structural variations can be reliably identified using single-cell sequencing, the genome-wide assessment of mutations in single cells is still challenging due to artifacts introduced by whole genome amplification [49]. One potential use of single-cell sequencing may lie in the characterization of CTCs (see below); it remains to be determined, however, whether CTCs are representative of the whole tumor and if those cells are likely to constitute the ones that mediate the metastatic progression of cancers [50].

### *In situ* topological genotyping

Given the spatial heterogeneity in tumors, methodologies that allow for *in situ* topological genotyping, assessment of gene copy number aberrations, and expression and/or activation of the protein products of genes targeted by genetic hits provide detailed geographical information for the inference of clonal structure and tumor topology that is complementary to that provided by bulk and single-cell sequencing. Techniques routinely employed in pathology laboratories, including *in situ* hybridization and immunohistochemistry, using robust methods and validated antibodies, have been successfully employed not only to unravel intra-tumor genetic heterogeneity in cancers [38,39] but also to provide important information on the expression and activation of genes targeted by gene copy number aberrations (for example, gene amplification) and mutations or their downstream targets. Furthermore, a combination of FISH and immunohistochemistry has proven useful in the development of computational models to infer tumor growth patterns and evolutionary dynamics, and also in the characterization of the interactions between intra-tumor heterogeneity and pathological complete response in breast cancers [51]. Novel techniques, including the synchronous *in situ* detection of multiple expressed mutations, based on padlock probes and *in situ* target-primed rolling-circle amplification [52], have also been successfully applied to the detection of somatic point mutations, the discrimination between members of a gene family and for multiplex detection of transcripts in human and mouse cells and tissue. In addition to multiplex geographical genotyping of tumors, *in situ* analysis of mRNA has a detection resolution that may allow for the study of allele-specific expression directly on tissue samples. These features render the approaches described above an exciting complement to both bulk and single-cell sequencing genomics, where genetic aberrations found in bulk or single cells can be traced back to the tumor topology. This integrative approach would not only define regions of dominance of specific driver genetic events, but also provide an integrated view of the potential functional consequences of these genetic aberrations on gene and protein expression, and pathway activation.

## Assessing intra-tumor heterogeneity: clinical applications

It is undeniable that a high degree of phenotypic and genetic intra-tumor heterogeneity exists in breast tumors, and that this phenomenon may have a direct impact on both diagnosis and disease management. Given the discordance in the mutational repertoire between primary tumor and metastatic lesions within the same patient [6,8], a single biopsy is unlikely to represent accurately the genomic landscape of a patient’s cancer. On the other hand, serial tumor sampling at crucial time points (for example, development of metastatic disease or progression after initial response to systemic therapy) may help monitor the temporal heterogeneity [12]. In many cases, however, multiple sampling is not clinically feasible.

Tumor specimens are routinely formalin-fixed paraffin-embedded (FFPE) to preserve their histology, and these currently represent the largest source of clinical material but are underused in large scale whole exome or whole genome studies due to technological challenges. The ability to perform MPS on DNA and RNA material extracted from FFPE tissues will greatly increase the availability of material amenable to the characterization of intra-tumor heterogeneity. Currently, targeted sequencing using capture panels for DNA derived from FFPE samples allows the detection of clonal and subclonal mutations [53]. While the technology is improving such that routine whole-exome sequencing will soon be possible, translating single-cell methods into the clinic using FFPE material presents a bigger challenge.

**Figure 3:**
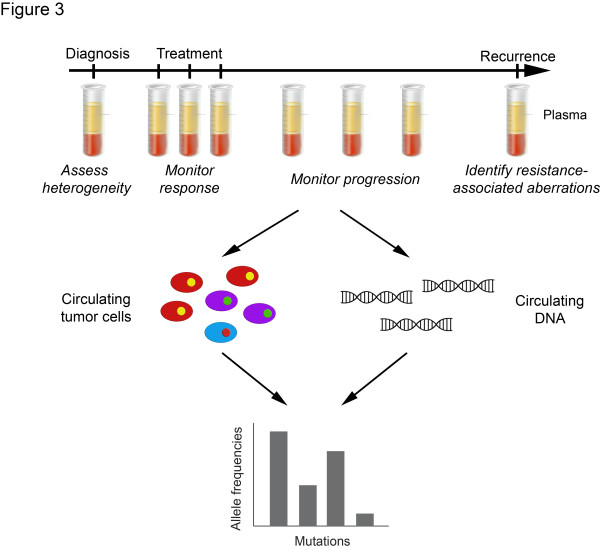
**Circulating tumor cells and circulating cell free DNA.** Circulating tumor cells and cell free DNA, which is at least in part derived from tumor cells, are obtained through serial collection of blood samples. Genetic analysis of these samples may provide information about the genetic heterogeneity of a cancer, and enable monitoring of its response to therapeutic interventions, the progression of the disease, and the emergence of resistant clones.

As CTCs and circulating cell-free plasma DNA (cfDNA) are likely to originate from tumor cells and thus correlate with disease burden and may overcome sampling bias, blood biomarkers may serve as a surrogate for spatial heterogeneity and as a marker for temporal heterogeneity (Figure 3). The accurate detection and molecular profiling of CTCs remain obstacles to using CTCs as a surrogate for heterogeneity. In particular, the most popular CTC capture method, CellSearch, relies on the detection of the surface epithelial marker EpCam; it is unclear, however, whether all CTCs from epithelial tumors express EpCam and are detected by this methodology [54]. Furthermore, even in metastatic breast cancer patients, a median of only five CTCs are detected per 7.5 ml blood [50]. Given the rarity of CTCs and the high possibility of contamination with non-tumor cells, the molecular profiling technique used for their analysis needs to be remarkably sensitive to be able to detect the tumor-specific aberrations. As a surrogate for heterogeneity, whether CTCs are truly representative of the entire tumor or of more genetically advanced clones remains to be seen.

In contrast to the challenges faced in the molecular profiling of CTCs, the profiling of cfDNA requires sequencing and bioinformatic methods that are capable of detecting ultra-rare aberrations in fragmented DNA. The recent proof-of-principle study in which temporal heterogeneity was traced using specific mutations in *PIK3CA* and *TP53* in cfDNA from breast cancer patients suggests that it is a viable, sensitive and real-time surrogate for tumor burden [55]. That study, however, was limited to metastatic cancers and whether cfDNA would constitute an optimal marker for patients with early-stage breast cancer is currently unknown. Furthermore, the specific mutations were detected in only 30 of 52 patients, suggesting that, in its current form, its clinical utility is uncertain. It is plausible that in the remaining patients the proportion of cfDNA is below the detection of conventional MPS methods, arguing for more sensitive technologies.

In addition to monitoring progression using specific mutations, *de novo* identification of aberrations in cfDNA may help in assessing the overall heterogeneity and identifying genetic aberrations responsible for drug resistance. Although it has recently been reported that exome sequencing of plasma DNA is possible and that many of the mutations identified are concordant between plasma and synchronous metastasis biopsies, in two of the cases studied, 38.4% and 80.7% of the mutations were found in either only the plasma or the metastasis [56]. In particular, in one case, 76.6% of the mutations were found only in the metastasis with allele frequencies of <20% [56], suggesting that at the sequencing depth employed using the standard sequencing methods, plasma DNA was not a comprehensive representation of the overall intra-tumor heterogeneity. The use of high-fidelity high-depth sequencing methods may increase the sensitivity for the detection of subclonal mutations present in tissues and plasma.

## Conclusion

The realization that tumors are composed of several subclones of tumor cells that, in addition to the founder genetic events, harbor private mutations, some of which constitute *bona fide* driver genetic aberrations, has resulted in a paradigm shift in regards to our understanding of cancers. Understanding the concept of intra-tumor genetic heterogeneity is providing answers to clinical questions that had historically baffled oncologists, pathologists and scientists. By the same token, the degree of genetic heterogeneity observed within cancers is undoubtedly a daunting observation from the precision medicine standpoint. We would argue, however, that by comprehensively cataloguing this phenomenon and identifying the mechanisms that result in its emergence, novel approaches to targeting cancers may emerge. In fact, recent advances in next generation sequencing analysis have opened up new possibilities for the way we approach the problem; despite the current statistical and technological challenges that have yet to be resolved, the opportunities are enormous.

## Abbreviations

cfDNA: Cell-free DNA; CGH: Comparative genomic hybridization; CSC: Cancer stem cell; CTC: Circulating tumor cell; DCIS: Ductal carcinoma *in situ*; FFPE: Formalin-fixed paraffin-embedded; FISH: Fluorescence *in situ* hybridization; MPS: Massively parallel sequencing; PCR: Polymerase chain reaction.

## Competing interests

The authors declare that they have no competing interests.
